# Precise Stepwise Synthesis of Donor-Acceptor Conjugated Polymer Brushes Grafted from Surfaces

**DOI:** 10.3390/ijms23116162

**Published:** 2022-05-31

**Authors:** Anna Grobelny, Artur Grobelny, Szczepan Zapotoczny

**Affiliations:** 1 Faculty of Chemistry, Jagiellonian University, Gronostajowa 2, 30-387 Kraków, Poland; grobelnyania@gmail.com; 2 Selvita Services Sp. Z o.o., Bobrzyńskiego 14, 30-348 Kraków, Poland; artur.grobelny@selvita.com

**Keywords:** donor-acceptor polymers, polymer brushes, cross-coupling reactions, low-bandgap polymers

## Abstract

Donor-acceptor (D-A) conjugated polymers are promising materials in optoelectronic applications, especially those forming ordered thin films. The processability of such conjugated macromolecules is typically enhanced by introducing bulky side chains, but it may affect their ordering and/or photophysical properties of the films. We show here the synthesis of surface-grafted D-A polymer brushes using alternating attachment of tailored monomers serving as electron donors (D) and acceptors (A) via coupling reactions. In such a stepwise procedure, alternating copolymer brushes consisting of thiophene and benzothiadiazole-based moieties with precisely tailored thickness and no bulky substituents were formed. The utilization of Sonogashira coupling was shown to produce densely packed molecular wires of tailored thickness, while Stille coupling and Huisgen cycloaddition were less efficient, likely because of the higher flexibility of D-A bridging groups. The D-A brushes exhibit reduced bandgaps, semiconducting properties and can form aggregates, which can be adjusted by changing the grafting density of the chains.

## 1. Introduction

Donor-acceptor polymers (D-A) are macromolecules with an alternating distribution of electron donor and electron acceptor moieties along the backbone. Since their introduction in 1993 by the Havinga group [1], they have gained great interest as a particular example of conjugated polymers (CP). CPs are attractive for various photovoltaic or electronic applications [2] due to their unique electrical [3] and photophysical [4] properties, as well as their ease of processability [5], flexibility [6], low-cost [7] or a broad range of available architectures and chemical structures. One of the desired features of CP for such applications, typically provided by D-A polymers, is a low optical bandgap, which determines the efficient transport of charge carriers by promoting intramolecular charge transfer [8]. Another advantage of low-bandgap D-A copolymers is their high absorption coefficients in longwave regions [9] (when compared to conjugated homopolymers, such as P3HT (poly(3-hexylthiophene)) [10]), leading to efficient harvesting of photons in visible and near-infrared ranges [11].

The application of a D-A architecture is one of the most used approaches to obtain macromolecules with a narrow optical bandgap. Reduction of the energy gap width is possible by proper selection of donor and acceptor mers characterized by adequate HOMO and LUMO levels [12]. The value of the HOMO level depends mainly on the electron-rich moieties, while the electron-poor molecule has a greater contribution to the energy of LUMO [13]. Furthermore, assignment to a donor or acceptor role is not obvious because it depends on the relationship between both molecules, contributing to the polymer chain [14] and their donating and accepting properties could be modified by the incorporation of proper substituents [12]. Polymer chains built from strong electron donors and acceptors exhibit low ionization potential and high electron affinity, enhancing the injection of both charge carriers, thus the possibility of ambipolar charge transfer [14]. The periodicity reflected in an alternating arrangement within D-A polymer architecture also affects material crystallinity and self-organization, which can enhance the mobility of electrons and resistance to oxygen or water [15]. Among a wide range of monomers used as donor molecules for the synthesis of D-A polymers, those based on thiophene are characterized by efficient light absorption, high stability and hole transport properties. Benzothiadiazole derivatives are an example of preferred chromophores with acceptor properties that exhibit a desired planar geometry [16].

The majority of devices constructed with CP demand the fabrication of conductive layers deposited on proper substrates [2]. The building process of such systems usually comprises, first, the synthesis of macromolecules in solution, followed by their deposition on a surface [17]. The solubility of CP, necessary to process them, is usually ensured by the incorporation of various substituents into the macromolecules [18], mainly bulky alkyl chains [19]. Nevertheless, typical CP films consist of entangled chains that hinder electron transport and can cause scattering and loss of charge carriers [20]. Moreover, due to the lack of covalent attachment of such a layer to the substrate, they are prone to delamination [21]. The above-mentioned problems could be addressed by surface-grafted polymer brushes (PB), which consist of macromolecules attached to the substrate by one end at a grafting density high enough to provide stretched conformations of the chains [22]. In addition, thanks to the ordered structure of conductive PB, with macromolecules oriented perpendicularly to the surface, they may provide a directional flow of electrons. As a result, conductive PB were shown to exhibit, e.g., three orders of magnitude higher current density [20] and enhanced stability [23] in comparison to the same polymers obtained in solutions. A well-defined arrangement of polymer chains within the layer also contributes to strengthening inter and intramolecular interactions, as they are both dependent on the macromolecular organization [24]. That is why a lot of effort has been put into investigating conformational changes in grafted macromolecules, which can be induced by external stimuli [25]. Furthermore, PB can coat substrates of various shapes and chemical compositions. All these unique features make them attractive candidates for application in, e.g., solar cells, [26] OLEDs (Organic Light Emitting Diodes) [27], light-harvesting [28], sensors [29], and drug delivery [30].

PB can be obtained via two main approaches. The “grafting to” approach is based on the synthesis of macromolecules with active end-groups in a solution, followed by coupling them to the desired surface with proper reactive groups. In the case of the “grafting from” methodology, the initiator monolayer is formed first, and then polymer chains are grown from the modified surface via various polymerization techniques [31]. Despite the simplicity of the “grafting to” method and the possibility of detailed characterization of macromolecules synthesized in solutions [32], it is less effective for the formation of thick PB with high grafting density due to steric hindrance of the already grafted chains blocking the adsorption of new ones.

Although the “grafting from” methodology is compatible with many polymerization techniques, such as ATRP (Atom Transfer Radical Polymerization) [33], RAFT (Reversible Addition-Fragmentation Chain Transfer Polymerization) [26] or PIMP (Photoiniferter-Mediated Polymerization) [34], facile synthesis of surface-grafted conjugated PB is still challenging. They could be obtained by, e.g., Surface-Initiated Kumada Catalyst Transfer Polycondensation (SI-KCTP) [35] or oxidative polymerizations [36], usually applying thiophene-based monomers. A self-templating surface-initiated polymerization approach was also proposed, which allowed the formation of conjugated PB containing polyacetylene [23,37] or polythiophene [33,34] chains. Nevertheless, no such approaches were shown to lead to alternating D-A copolymer brushes. In solution, the synthesis of such polymers was performed mainly using transition metal-catalyzed cross-coupling, such as Stille [12], Sonogashira [38], Suzuki [38] or Huisgen reactions [39]. However, while homopolymer conjugated surface-grafted PB are more common in the literature [40,41], there are only a few reports on the synthesis of D-A polymers grafted to the surface [42,43,44]. Using the “grafting to” approach to attach chains obtained in the solution is also not trivial, as it requires the synthesis of polymers soluble in some solvents. To ensure this necessary solubility, monomers are usually derivatized with some bulky substituents, such as alkyl chains [45], but such modifications can significantly influence the properties of the material [46].

Here, we present a synthetic approach leading to conjugated surface-grafted D-A alternating copolymer brushes with a reduced optical bandgap. Appropriate donor and acceptor molecules were selected on the basis of the DFT (Density Functional Theory) calculations, which aimed at designing structures with narrow optical bandgaps. The monomers were then synthesized and applied in a surface-confined stepwise deposition approach using Stille, Sonogashira couplings and Huisgen cycloaddition (click) reaction. The proposed methodology enabled the formation of surface-grafted copolymer chains without the need to use bulky side groups. The growth of the PB from the surface was followed by spectroscopic ellipsometry and confirmed by contact angle measurements, atomic force microscopy (AFM), IR and UV-Vis spectroscopy, which additionally provides insight into interactions within the synthesized brushes. The influence of the grafting density on polymer growth was also investigated. As a result, the Sonogashira coupling approach was shown to be the most efficient in the synthesis of surface-grafted macromolecules that was ensured by a proper chain design. Finally, the conductivity measurements performed with conductive AFM revealed the semiconducting character of chains with an acetylene bridge.

## 2. Results and Discussion

### 2.1. Selection of Donor and Acceptor Moieties

Donor and acceptor monomers for the synthesis of low bandgap D-A PB were selected based on the results of DFT calculations of the alternating oligomers composed of those monomer units. The energy gap was estimated based on the energy of HOMO → LUMO vertical transition, according to the procedure described by Fernández-Gómez et al. [47] (see details in Section 3). The application of M06-2X functional was shown to provide a good correlation between the calculated optical bandgaps and experimental data for various conjugated copolymers composed of aryl units linked with an acetylene linker. 

**Scheme 1:**
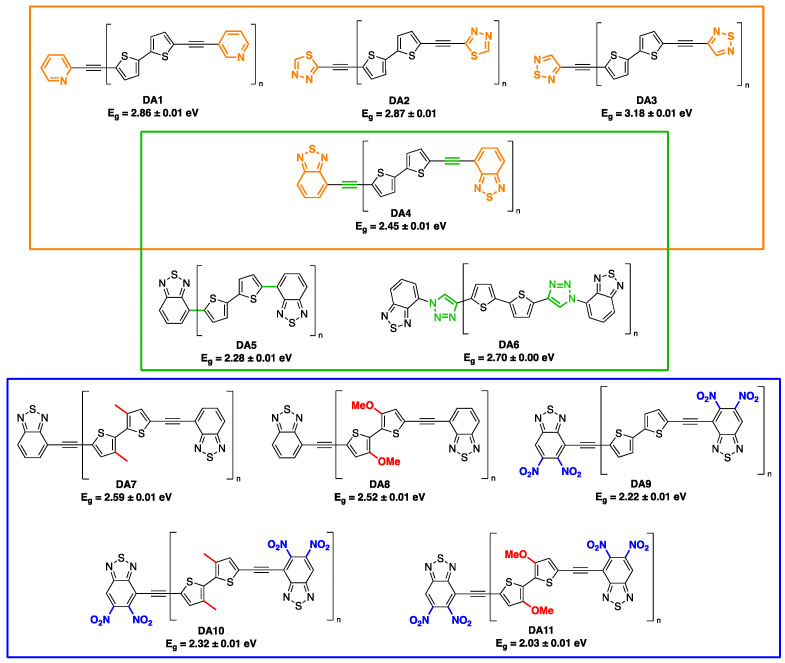
Calculated bandgaps of various D-A polymer structures differing in acceptor moiety (orange box), linking unit (green box) and substituents (blue box).

Thiophene-based monomers are frequently applied as donor moieties. A bithiophene core was selected, taking into account also its size—large enough to easily follow the thickness changes after its coupling to a surface but not significantly enhancing the steric hindrance. Four moieties were investigated as potential electron acceptors: pyridine [48], benzo[c][1,2,5]thiadiazole [17], 1,2,5-thiadiazol [49] and 1,3,4-thiadiazol [50] (DA1-4 in the orange box in Scheme 1). Within the tested alternating copolymer structures, the application of benzothiadiazole as an acceptor unit seemed to ensure the lowest value of the optical bandgap, so it was used in further steps of the design process. 

Next, the role of the linking unit between bithiophene and benzothiadiazole was tested. In this case, the choice of triple bond, single bond and 1,2,3-triazole ring was a result of the planned synthetic paths: Sonogashira, Stille and Huisgen reactions, respectively (**DA4**–**6** in the green box in Scheme 1). Surprisingly, the **DA5** polymer with a direct connection between aryl rings exhibits a narrower energy gap equal to 2.28 ± 0.01 eV than poly(aryleneethynylene) **DA2** with an acetylene bridge in the structure, despite the less rigid and planar geometry [51]. The higher value of the bandgap for **DA6** could be caused by the disturbed conjugation in the triazole ring.

The last step in the design of monomers was the selection of appropriate electron-withdrawing and/or electron-donating substituents, which could significantly affect the electronic properties of both monomers [12]. Due to the key role of proper adjustment of the energy levels of the donor and acceptor mers [14], the molecules were tested in various possible configurations. Methyl, methoxy and nitro groups were chosen to derivatize the core compounds (**DA7**–**11** in the blue box in Scheme 1). The **DA11** polymer, composed of 3,3′-dimethoxy-2,2′-bithiophene and 5,6-dinitrobenzo[c][1,2,5]thiadiazole, revealed the lowest bandgap of 2.03 ± 0.01 eV. Nevertheless, it is worth noticing that the presented values could be slightly overestimated, as the computed energy of vertical transition is a sum of adiabatic excitation and vibrational components. Thus, in addition to the values of the optical gaps, they also contain a contribution from the first or higher-order vibrational states [52].

The proper selection of monomers for the synthesis of low-bandgap polymers was confirmed by the estimation of the energy of HOMO and LUMO for all of the donor and acceptor moieties tested. The performed calculations revealed the best adjustment between the energy levels for both monomers that contributed to **DA11** since 3,3′-dimethoxy-2,2′-bithiophene exhibits the highest energy of HOMO and 5,6-dinitrobenzo[c][1,2,5]thiadiazole exhibits the lowest energy of LUMO among all molecules tested (Table 1).

### 2.2. Synthesis of Monomers

**Scheme 2:**
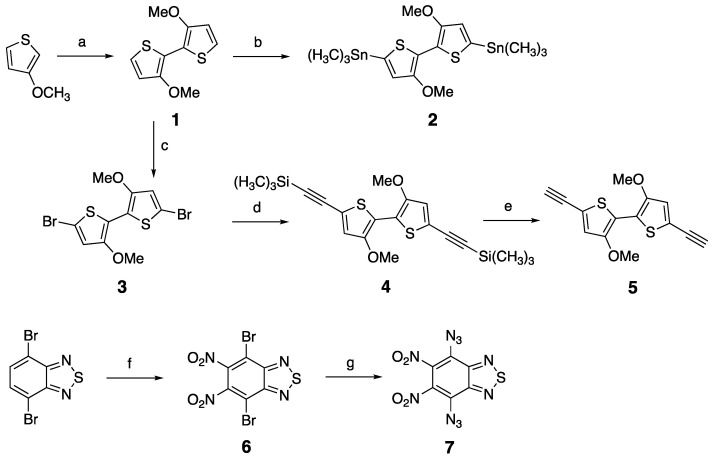
Synthetic paths for donor and acceptor monomers. Reaction conditions: (a) (1) *n*-BuLi, THF, −78 °C, 2 h; (2) Fe(acac)_3_, RT, 12 h; (b) (1) *n*-BuLi, THF, −78 °C, RT, 30 min; (2) Sn(CH_3_)_3_Cl, THF, −78 °C, RT, 12 h; (c) NBS, THF, RT, 10 min; (d) trimethylsilylacetylene, Pd(PPh_3_)_4_, CuI, *i*-Pr_2_NH, THF, 60 °C, 12 h; (e) K_2_CO_3_, MeOH, RT, 4 h; (f) HNO_3_, CF_3_SO_3_H, 0 °C, RT, 12 h; (g) NaN_3_, H_2_O, DMF, 0 °C, RT, 1 h.

The selected donor and acceptor molecules were then synthesized in a multistep procedure (Scheme 2). They were derivatized with proper functional groups, which enabled the application of the chosen coupling reactions to obtain D-A systems. The first target donor is 3,3′-dimethoxy-5,5′-diethynyl-2,2′-bithiophene **5,** equipped with two acetylene groups that are active in the Sonogashira and Huisgen reactions. It was synthesized starting with the coupling of 3-methoxythiophene rings in the presence of Fe(acac)_3_. The obtained compound **1** was then brominated in a typical process with NBS to perform the Sonogashira reaction with trimethylsilylacetylene, followed by deprotection of TMS with K_2_CO_3_ in the last step. In the case of Stille coupling, the final donor monomer **2** was synthesized by modification of intermediate **1** with Sn(CH_3_)_3_Cl.

The selected 5,6-dinitrobenzo[c][1,2,5]thiadiazole-based acceptor moiety was prepared in a classical nitration reaction of 4,7-dibromobenzo[c][1,2,5]thiadiazole, giving acceptor unit **6**, whose halogen substituents allow polymer brushes to be obtained via Sonogashira and Stille coupling. The bromine atoms of compound **6** were then substituted with azide groups in the S_N_Ar reaction to obtain monomer **7** for the Huisgen reaction. The structure of the desired donor and acceptor moieties, as well as all intermediate products, were confirmed by ^1^H and ^13^C NMR spectroscopy, HRMS analysis (see details in the SI Experimental Section), as well as by measured FTIR spectra (Figures S1–S4). 

### 2.3. Synthesis of Grafted D-A Polymer Brushes by Sonogashira Coupling

**Scheme 3:**
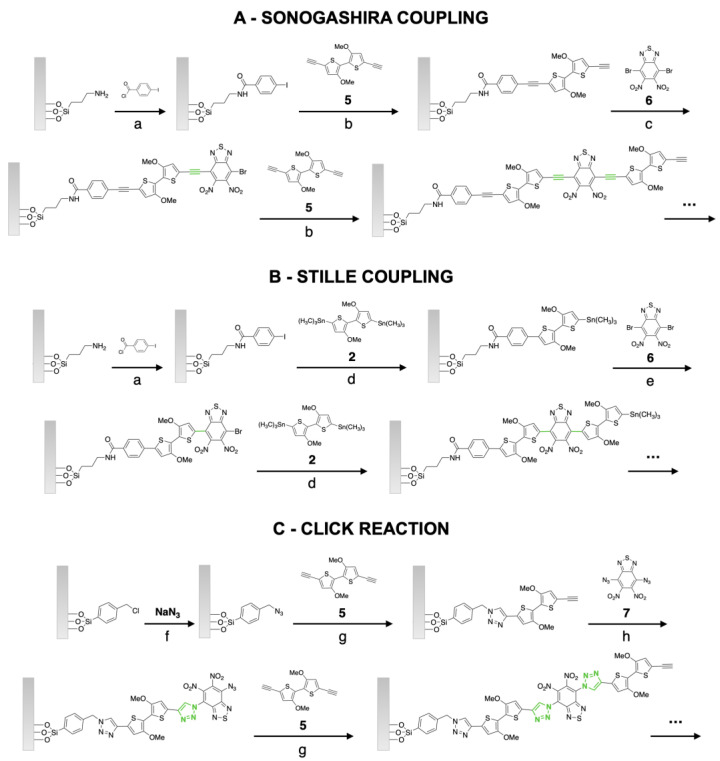
Synthesis of donor-acceptor polymer brushes via: (**A**), Sonogashira coupling, (**B**), Stille coupling, (**C**), Click reaction. Reaction conditions: (a) 4-iodobenzoyl chloride, TEA, DCM, RT, 12 h; (b) donor **5**, Pd(PPh_3_)_4_, CuI, DIPEA, DMSO, 90 °C, 12 h; (**c**) acceptor **6**, Pd(PPh_3_)_4_, CuI, DIPEA, DMSO, 90 °C, 12 h; (d) donor **2**, Pd_2_(dba)_3_, P(*o*-tol)_3_, toluene, RT, 2 h; (e) acceptor **6**, Pd_2_(dba)_3_, P(*o*-tol)_3_, toluene, 110 °C, 12 h; (e) NaN_3_, DMF, RT, 48 h; (f) donor **5**, CuI, DMSO, 40 °C, 2 h; (g) acceptor **7**, CuI, DMSO, 40 °C, 12 h.

**Figure 1:**
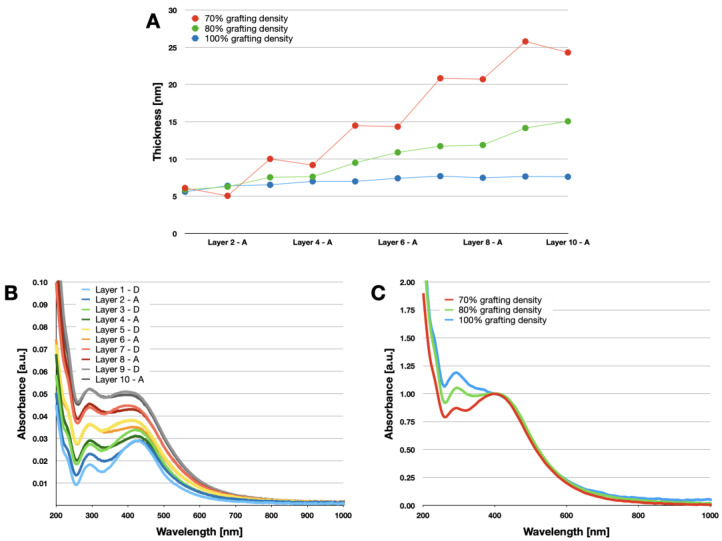
Characterization of D-A polymer brushes obtained via Sonogashira coupling reaction: (**A**) thickness of the material with different grafting density on silica substrate, (**B**) UV-Vis spectra after deposition of each layer for a sample with 80% grafting density on quartz substrate, (**C**) normalized UV-Vis spectra of the (D-A)_5_ brushes with various grafting density on quartz substrate.

In order to grow D-A chains from surfaces, the ITO, silica and quartz plates were first treated with a plasma cleaner to generate hydroxyl groups, which enabled the chemosorption of proper linker molecules. For the synthesis of polymer brushes via Sonogashira coupling, the surface was modified with APTES molecules (Scheme 3A). The deposition lasted only 2 h to avoid the formation of multilayers [53] and was followed by vacuum drying for 12 h [54], which resulted in a reasonable thickness of 1.6 nm as determined by spectroscopic ellipsometry. Then, aryl halide was introduced by the formation of the amide bond between APTES and 4-iodobenzoyl chloride. In this way, the iodide-terminated monolayer was formed, which was necessary to perform further reactions with the donor moiety equipped with triple bonds or tin substituents. First, polymer brushes were obtained in a stepwise alternating reaction of a proper donor and acceptor compounds via Sonogashira coupling. The course of the entire process was followed by spectroscopic ellipsometry, which revealed an increase in the thickness of the formed polymer layer (Figure 1A). Nevertheless, the observed growth of the formed starting monolayer (blue dots) was not linear, reaching the plateau value that could be caused by the limited efficiency of Sonogashira coupling under such confined surface conditions [55]. It should be emphasized that an incomplete reaction during the deposition of each layer (e.g., donor) would exclude the possibilities of bond formation in the next step, as no appropriate active groups could be exposed to the surface. Steric hindrance of neighboring chains hiding the active groups on the modified substrate could further affect the surface reactions. Another reason for the nonlinear growth of the brushes could be related to the formation of loops by the bifunctional monomer molecules reacting with two neighboring surface-tethered chains, disabling their further growth in the next steps. 

The progress of PB growth was also investigated by UV-Vis spectroscopy, which revealed an increase in absorption after the attachment of each bilayer (Figure 1B). Such an observation could be related to the growth of the thickness of the PB layer, as according to the Beer–Lambert law, the increase in the number of absorbing species should imply an increase in the total absorbance. Surprisingly, such an effect was observed after the reaction with the donor monomer but not the acceptor one. Nevertheless, the molar extinction coefficient, ε, of D-A polymers may vary with the ratio of the number of donor and acceptor units in a macromolecule, and it seems that the electron-withdrawing mers decrease the value of ε. The UV-Vis spectra are also often used to estimate the effective conjugation length in conductive polymers, as the absorption maximum exhibits a bathochromic shift with the increasing number of conjugated bonds [34]. In the case of D-A PB prepared via Sonogashira coupling, this effect could contribute to the widening of the band with the maximum at ca. 420 nm—the absorption tail was shifted toward a longer wavelength after each deposition step. The increase in the PB thickness also causes a relative increase in the intensity of the band near 300 nm when compared to absorption maximum visible at wavelengths above 400 nm. Such variations observed in the UV-Vis spectra could also be a superposition of intramolecular and intermolecular interactions, described as J-coupling and H-coupling, respectively. Both can be observed in highly ordered conjugated systems as a result of the parallel orientation of the monomer’s transition-dipole moments (TDM) along the polymer chain, and the antiparallel interference of the individual TDMs between two chains placed close to each other. In consequence, they induce contradictory changes in UV-Vis spectra: J-aggregates lead to a batochromic shift, while H-aggregates move the absorption maximum towards a shorter wavelength. Despite an opposite influence on the spectroscopic behavior of the coating, these two types of interactions are not competitive—they may be both enhanced by the extended, planar conformation of the chains [24]. Thus, the observed widening of the band and changes in intensity ratio could be assigned to the existence of both effects indicating the presence of ordered densely packed polymer brushes. The electronic absorption spectrum also enabled the estimation of the value of the direct optical bandgap as 2.34 eV, based on the Tauc plot method (Figure S6) [56].

**Figure 2:**
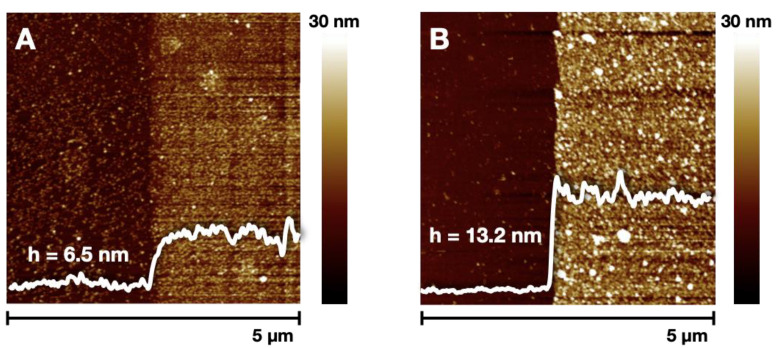
AFM pictures of polymer brushes with 80% grafting density on a silica substrate obtained via Sonogashira coupling and their thicknesses determined at the edges of the layers: (**A**) after deposition of 5 layers, (**B**) after deposition of 10 layers.

The changes in surface hydrophobicity after the addition of each layer were also investigated (Figure S7). The alternating rise and fall of the contact angle values during the whole process indicate the efficient attachment of each layer and the regular growth of D-A PB. The presence of a uniform organic film (surface roughness, R_a_ = 2.6 nm) on the silica substrate was also confirmed by atomic force microscopy (Figure 2). The captured images revealed that the brush thickness increased from 6.5 ± 0.5 to 13.2 ± 0.3 nm when comparing 5 and 10-layer-systems (80% grafting density; see further). The presented fabrication process consisted of 10 immersion steps, as the conducted DFT calculation revealed that oligomers created from 5 pairs of donors and acceptor moieties, (D-A)_5_, reach a plateau in the bandgap value, reflecting the Eg of even longer polymer chains. The IR spectra registered after a single donor and subsequent acceptor coupling also indicate proper bond formation between both molecules on a surface (Figure S8). For the sample with the donor **5** monolayer, the bands around 2200 and 3300 cm^−1^, assigned to the vibrations of the C≡C−H group, were noticed, while after the coupling of acceptor **6**, these characteristic bands disappeared due to the terminal C≡C group turning into an internal one.

#### 2.3.1. Varying the Grafting Density

The influence of the grafting density on the polymer growth was studied to reveal possible limitations of the surface-confined reactions. The mixtures of APTES and ClPTES were deposited on the surface to exchange some active amine groups of APTES for inactive chlorine in ClPTES; as such, halogens do not react with 4-iodobenzoyl chloride, and polymer brushes cannot grow from those spots. Four grafting densities were tested with 100%, 75%, 50% and 0% APTES in the feed APTES/ClPTES solution. The actual composition of the mixed monolayers was determined by obtaining the N:Cl ratio using XPS (Table S1). Then, the D-A chains with different grafting densities were prepared using the same procedure. Decreasing the APTES content on the surface from 100% (full APTES monolayer) to 80% allowed the thickness of the (D-A)_5_ brushes to double, which reached ca. 25 nm when the APTES content was decreased to 70% (Figure 1A). 

Thus, it seems that the increase in distances between neighboring active groups on a surface promotes the growth of the brushes, likely due to hindered formation of the mentioned loop structures and limited steric hindrance of the chains. In the case of the sample with the lowest studied grafting density (70%), changes in thickness (by ellipsometry) observed after each attachment of the acceptor monolayer were negative when compared to the binding of the donors. Such observations could be attributed to the smaller size of the acceptor molecule, as well as to conformational changes resulting in better packing of the acceptor-ended chains. Complete elimination of APTES from the initiator monolayer resulted in no polymer formation, which points to the necessity of particular surface groups for the reaction to proceed. The modification of the grafting density also induced changes in the UV-Vis spectra (Figure 1C, compare the spectra of the monomers in Figure S9). They revealed a drop of the absorbance ratio of the bands with maximum at 300 nm and 420 nm (A_300_/A_420_) with the increasing grafting density. Such spectroscopic behavior could be assigned to the decreasing contribution of H-type aggregates caused by the insufficient proximity of the chains.

#### 2.3.2. Conductivity Measurements

**Figure 3:**
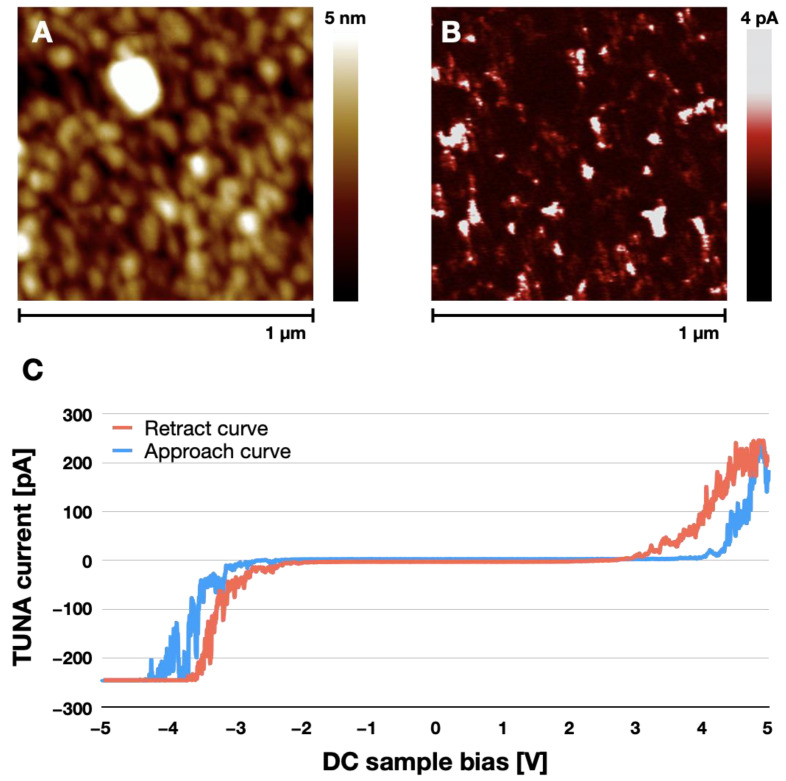
AFM images visualizing: (**A**) topography, (**B**) conductivity of polymer brushes obtained via Sonogashira coupling on the ITO substrate, (**C**) a representative I/V curve.

The conductivity of synthesized polymer brushes was investigated using conductive atomic force microscopy (AFM). In order to avoid the possibility of direct electrical contact of the AFM tip with the ITO surface, a sufficiently thick (13.9 ± 0.5 nm) sample with 80% grafting density was characterized (Figure S10). The current map revealed relatively uniform conductivity of the organic layer, and the shape of the collected I/V curves indicated the semiconducting character of the synthesized brushes (Figure 3). The performed measurements allowed the estimation of the conductivity value at the level of 8∙10^−6^ S/cm (see details in SI Experimental Procedures). All these observations clearly indicate the formation of conjugated D-A chains during the Sonogashira process. 

### 2.4. Synthesis of Grafted D-A Polymer Brushes by Stille Coupling

The initiator moiety deposited for the Sonogashira coupling was also used to obtain D-A polymer brushes applying the Stille methodology. Due to the presence of aryl halide, it allows the reaction with proper tin derivatives of the bithiophene donor compound **2** to be performed, which could further bind the same acceptor unit **6** in the following step (Scheme 3B). The conducted spectroscopic ellipsometry measurements revealed the progressive increase in thickness of the organic layer (Figure S11). The observed growth was significantly smaller when compared to the PB obtained via the Sonogashira coupling, probably due to the lack of an acetylene bridge that influences the monomer size and stiffness of the formed chains. The less rigid structure of macromolecules, resulting from the presence of exclusively C-C single bond linkers, could also contribute to the distinct behavior of PB with various grafting densities. In that case, the thickest polymer layer was obtained for 80% grafting density. A further extension of the distance between neighboring chains could lead to less extended mushroom or even pancake conformations of the chains. [31] Deposition of the synthesized moieties was also observed in the AFM images, which revealed the presence of an organic layer with 5.0 ± 0.2 nm thickness after 10 steps (Figure S12). 

**Figure 4:**
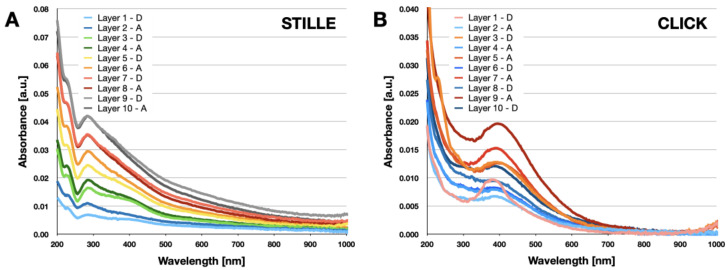
UV-Vis spectra of polymer layer obtained on quartz substrate via: (**A**), Stille coupling, (**B**), Click reaction.

The gradual growth of D-A PB via Stille coupling somehow induced different changes in the measured UV-Vis spectra (Figure 4A; compare the monomers spectra in Figure S13) as compared to the Sonogashira coupling (Figure 1B). The deposition of each bilayer caused an increase in the absorbance of the sample and resulted in the widening of the band to longer wavelengths due to increasing conjugation length. However, the shape of the spectra was noticeably different from those observed for Sonogashira brushes. An absorption band with a maximum near 400 nm was not very distinct (a shoulder), and the second one was shifted towards even shorter wavelengths (ca. 280 nm). Such differences should be the result of the absence of an acetylene bridge along the chains that resulted in a less rigid structure, leading to a smaller effective conjugation length. The single-bond linker between both moieties also influences the planarity within the chains. Furthermore, such differences in polymer conformation impact the strength of interactions within and between macromolecules since the formation of J and H-type aggregates depends on the arrangement within the whole system. Therefore, the observed single broad band with a long tail without discrete structure could suggest the contribution of chains of various conjugation lengths and weaker aggregation ranges due to a less rigid structure. The lack of an evident band in the spectrum hindered the estimation of the optical bandgap, which, based on the Tauc method, revealed a value of 2.99 eV (Figure S14).

### 2.5. Synthesis of Grafted D-A Polymer Brushes by Click Reaction

To check the possibility of formation of D-A PB via Huisgen reaction, the (p-chloromethyl)phenyltrimethoxysilane was deposited, whose chloride atom was substituted by the azide group in the S_N_2 process. The introduction of the -N_3_ moiety enables further bonding of donor moiety **5** with the creation of a triazole link according to a well-known click mechanism. The polymer chains were then prolonged with acceptor moieties **7** thanks to the presence of the azide substituent (Scheme 3C). Then, PB with various grafting densities were obtained using the mixed initiator monolayer approach. 

In that case, trimethoxy(p-tolyl)silane as an inactive specie was used because of the absence of a chloride atom in the structure, it cannot react with sodium azide, as well as donor and acceptor monomers. Changes in the initiator layer composition were confirmed by decreasing the chloride surface content in XPS spectra (Table S2). 

The growth of the grafted macromolecules was followed by spectroscopic ellipsometry, which reveals the positive impact of reducing the grafting density, as shown in previous examples (Figure S15). Looking into the details, it seems that the most regular growth of polymer chains was observed in the case of the sample with the smallest grafting density. Such behavior, like those observed for Sonogashira brushes, could be a result of a smaller steric hindrance and limited possibility of the loops formation. Moreover, it indicates quite a stiff structure of the synthesized macromolecules that prevents adopting mushroom or pancake conformations by the chains due to the presence of bridging triazole rings. Surprisingly, a high efficiency assigned to click chemistry was not reflected in the obtained data, as the thickness of the (D-A)_5_ brushes does not exceed the thickness of PB obtained in palladium-catalyzed processes.

The performed contact angle measurements revealed the expected, alternating changes in surface hydrophobicity after deposition of each layer, as in the case of the brushes formed in Sonogashira coupling (Figure S16). The collected AFM images also suggest the proper course of the process and confirm the presence of an organic layer with 8.6 ± 0.7 nm thickness after the deposition of 10 layers (Figure S17).

According to the measured UV-Vis spectra, the observed changes were different from those noticed for the bushes obtained in previous cross-coupling processes (Figure 4B; compare the monomers spectra in Figure S18). The absorbance does not increase after every two steps, but the band successively grows and broadens when the spectra collected separately after donor (or acceptor) deposition are taken into consideration. That is why it seems that the changes in the molar extinction coefficient after the introduction of the acceptor unit play a key role in this case. The changes in the spectra could also be explained by disturbed conjugation caused by the incorporation of triazole rings within the backbone. The type of linking unit is also significant for the strength of the interaction within the layer. The presence of one maximum around 400 nm suggests the predominant character of J-aggregation, and the widening of the band could be assigned to the creation of chains with various conjugation lengths. The estimated value of the optical bandgap equal to 2.38 eV (Figure S19) is lower than for the brushes obtained by Stille coupling (2.99 eV) but higher than the one of the Sonogashira-based brushes (2.34 eV), pointing to the importance of the rigidity of the polymer chains in designing low-bandgap brush systems.

## 3. Materials and Methods

### 3.1. Materials

(3-aminopropyl)triethoxysilane (APTES, 99%), (3-chloropropyl)triethoxysilane (ClPTES, 95%), 3-methoxythiophene (98%), chloroform-*d* (CDCl_3_, 99.8 atom % D), dimethyl sulfoxide-*d* (DMSO-*d*_6_, 99.9 atom % D), dimethyl sulfoxide (DMSO, anhydrous, ≥99.9), *N*,*N*-diisopropylethylamine (DIPEA, ≥99%), fuming nitric acid (HNO_3_, >99%), silica gel (high-purity grade, average pore size 60 Å (52–73 Å), 70–230 mesh, 63–200 μm, for column chromatography), sodium azide (NaN_3_, ≥99.5%), sodium sulfate (Na_2_SO_4_, anhydrous, granular, ≥99.0%), sulfuric acid (H_2_SO_4_, 95–97)%, toluene (anhydrous, 99.8%), triethylamine (TEA, ≥99.5%), trimethyltin chloride (Me_3_SnCl), tris(dibenzylideneacetone)dipalladium(0) (Pd_2_(dba)_3_, 97%) and tri(*o*-tolyl)phosphine (P(*o*-tol)_3_, 97%) were all purchased from Sigma-Aldrich (St Louis, MO, USA). Aluminum oxide (Al_2_O_3_, for chromatography, neutral, Brockmann I, 50–200 μm, 60 Å), *n*-butyllithium (*n*-BuLi, 2.5 M in hexane) and tetrahydrofuran (THF, 99.5%, Extra Dry over Molecular Sieve, Stabilized) were obtained from Acros Organics (Geel, Belgium). 4-iodobenzoyl chloride (95%), 4,7-dibromobenzo[c][1,2,5]thiadiazole (95%), copper(I) iodide (CuI, 98%), *N,N*-diisopropylamine (*i*-Pr_2_NH, 99%), iron(III) acetylacetonate (Fe(acac)_3_, 98%), *N*-bromosuccinimide (NBS, >98%), tetrakis(triphenylphosphine)-palladium(0) (Pd(PPh_3_)_4_, 98%), trifluoromethanesulfonic acid (CF_3_SO_3_H, 99%), trimethoxy(*p*-tolyl)silane (95.1%) and trimethylsilylacetylene (97%) were all ordered from Fluorochem Ltd. (Hadfield, UK). Ammonia (NH_3_, 30%, cz.d.a.), chloroform (CHCl_3_, p.a.), dichloromethane (DCM, p.a.), diethyl ether (Et_2_O, p.a.), ethanol (EtOH, p.a.), ethyl acetate (p.a.), hexane (p.a.), hydrogen peroxide (H_2_O_2_, 30%), methanol (MeOH, p.a.), *N,N*-dimethylformamide (DMF, p.a.), potassium carbonate (K_2_CO_3_, p.a.), sodium thiosulfate (Na_2_S_2_O_3_, p.a.), toluene (p.a.) and tetrahydrofuran (THF, p.a.) were obtained from Chempur (Piekary Slaskie, Poland). (*p*-chloromethyl) phenyltrimethoxysilane (95%) was purchased from ABCR (Karlsruhe, Germany). Acetonitrile (MeCN, spectrophotometric grade, ≥99.5%) was ordered from Honeywell (Morristown, NJ, USA). 

### 3.2. Methods

The NMR spectra of the synthesized compounds were recoded using an Avance II (300 MHz) or Avance III HD (400 MHz) spectrometer (Bruker, Santa Barbara, CA, USA). All presented data are referenced relative to the solvent residual signal of Chloroform-*d* (δ = 7.26 ppm in the case of ^1^H NMR and δ = 77.16 ppm for ^13^C NMR) or DMSO-*d*_6_ (δ = 2.50 ppm in the case of ^1^H NMR and δ = 39.52 ppm for ^13^C NMR). A Varian Cary 50 UV-VIS spectrometer (Agilent Technologies, Santa Clara, CA, USA) was applied to register the spectra of monomers solutions (transmittance mode, range: 200–800 nm, data interval: 1 nm, scan rate: 600 nm/min), as well as polymer brushes grafted from quartz substrate (transmittance mode, range: 200–1100 nm, data interval: 1 nm, scan rate: 150 nm/min). The contact angle measurements were performed using Surftens Universal equipment (OEG GmbH, Frankfurt, Germany). For every sample, at least 10 measurements were performed in 3 locations, and the presented results were calculated as arithmetic means of all measured values. The measurements of the thickness of polymer brushes on silica surface were performed using Spectroscopic Ellipsometer M200U (J. A. Woollam, Lincoln, NE, USA). The presented values were arithmetic means of at least 5 measurements fitted in CompleteEASE software to the Cauchy model in the range 550–1000 nm. A Dimension Icon Atomic Force Microscope (Bruker, Santa Barbara, CA, USA) working in a soft PeakForceTapping^®^ mode was applied to collect topography images using SCANASYST-AIR cantilever (nominal force constant 0.4 N/m). The PeakForce TUNA mode with a diamond-coated silicon probe (spring constants 5.8 N/m and tip radius 133 nm) was applied to perform conductivity experiments. The conductivity images were collected with the bias voltage of 3.5 V and a setpoint value corresponding to a maximum force of 100 nN. The I/V plots were obtained using a ramp mode in the range −5 to 5 V in 50 random places. To estimate the conductivity value, the linear part from −4 to −3 V was taken into consideration. All data were evaluated using NanoScope Analysis software (Bruker, Santa Barbara, CA, USA). XPS measurements were performed using a Prevac photoelectron spectrometer (Rogów, Poland) with a hemispherical analyzer VG SCIENTA R3000 at RT in anultrahigh vacuum. The monochromatic aluminum single anticathode source AlK_α_ was applied (E = 1486.6 eV). The obtained data were processed using CasaXPS Software (Casa Software LTd.). The Nicolet iS10 Spectrometer (ThermoFisher ScientificTM, Waktham, MA, USA) equipped with the ATR accessory (SMART iTX) and MCT/A detector was applied to register FTIR spectra (number of scans: 128, range: 650–4000 cm^−1^, resolution: 4 cm^−1^, gain: 2.0). The presented data were baseline corrected using OMNIC software. High-resolution mass spectra of synthesized compounds were recorded using MicrOTOF II (Bruker, Bremen, Germany) mass spectrometer with electrospray ionization source (ESI) or atmospheric pressure chemical ionization (APCI) and time of flight analyzer as a detector.

### 3.3. DFT Calculation

Gaussian09 software (Gaussian, Inc., Wallingford, CT, USA) was applied to conduct the theoretical calculations. The values of optical bandgaps for all oligomers were estimated according to the procedure described elsewhere [47]. Briefly, the energy of HOMO → LUMO vertical transitions was calculated by means of time-dependent density functional theory using Truhlar’s Minnesota functional (M06-2X model) with 6-31G(d) for oligomers from n = 1–5. Next, the optical bandgap Eg was estimated based on the formula:

E_n_ = E_∞_ + (E_1_ − E_∞_)e^−a(n−1)^

where: 

E_n_ is the energy of HOMO → LUMO vertical transition of oligomer composed of n mers;

E_∞_ is the excitation energy of the final polymer. 

The values of the HOMO and LUMO levels for all tested monomers were calculated using M06-2X DFT functional with 6-31G(d) base, as well as using the MP4(SDQ) level of theory with a 6–311 + G(d,p) base.

### 3.4. Substrate Preparation

All substrates were sonicated for 10 min in EtOH and dried under a stream of argon. Then, the silica and quartz samples were immersed in a solution of 30% H_2_O_2_ and H_2_SO_4_ (*v*:*v*, 1:3) for 15 min at RT; meanwhile, ITO plates were cleaned in a mixture of 30% H_2_O_2_ and 30% aq. NH_3_ (*v*:*v*, 1:1) for 60 min at 50 °C. Next, all slides were rinsed with copious amounts of water, THF and toluene. After drying under the stream of ambient gas, they were treated for 1 min with the plasma pen and placed for 30 min in the UV-Ozone Cleaner.

### 3.5. Preparation of the Initiator Monolayer for the Processes Based on Stille and Sonogashira Coupling

The initiator monolayer was prepared on the substrates in two steps. Firstly, all plates were immersed in 10 mL of toluene, and 50 μL of APTES (0.21 mmol) was added. After 2 h of reaction at RT, the samples were sonicated for 10 min in toluene, rinsed with copious amounts of DCM, dried under the stream of argon and placed in vacuo overnight. For the preparation of mixed monolayers instead of the APTES solution, the feed mixtures were prepared by mixing the solutions of APTES and inactive ClPTES in various ratios (0%, 25%, 50%, 75% of APTES) and applied the same way. Then, the modified plates were immersed in a solution of 11 μL of TEA in 9 mL of DCM and the mixture of 4-iodobenzoyl chloride (20.1 mg, 0.075 mmol) in 6 mL of DCM was added dropwise. The reaction was left to proceed overnight at RT, and all substrates were sonicated for 5 min in DCM, rinsed carefully with MeOH and finally dried under the argon stream.

### 3.6. Preparation of the Initiator Monolayer for the Process Based on Huisgen Cycloaddition (Click Reaction)

The substrates were placed in 10 mL of toluene containing 100 μL of (*p*-chloromethyl) phenyltrimethoxysilane (0.46 mmol). The reaction was kept overnight at RT, and then the plates were sonicated in toluene for 10 min, rinsed with a copious amount of DCM and dried under the stream of argon. For the preparation of mixed monolayers, the feed mixtures also contain inactive trimethoxy(*p*-tolyl)silane in various ratios (0%, 25%, 50%, 75% of (*p*-chloromethyl)phenyltrimethoxysilane) were prepared and applied the same way. Next, all samples were immersed in a solution of NaN_3_ (3.2 mg, 0.050 mmol) in DMF (10 mL) for 48 h. The cleaning of the substates was as follows: sonication in DMF, MeOH and water for 5 min each, rinsing with MeOH and drying under the ambient gas stream.

### 3.7. Sonogashira Coupling

The reaction mixtures for donor/acceptor deposition steps were prepared in pressure tubes heated under the argon flow. After cooling down to RT, the proper donor or acceptor compound was added: 3,3′-dimethoxy-5,5′-diethynyl-2,2′-bithiophene (**5**, donor) (41 mg, 0.15 mmol) or 4,7-dibromo-5,6-dinitrobenzo[c][1,2,5]thiadiazole (**6**, acceptor) (57 mg, 0.15 mmol), respectively. Next, the catalysts, Pd(PPh_3_)_4_ (4.25 mg, 0.0037 mmol), CuI (0.7 mg, 0.0037 mmol) were added into the tube, and all solids were dissolved in a mixture of anh. DMSO (10 mL) and DIPEA (5 mL). The substrate coated with the initiator monolayer was immersed in the prepared solution (in a glovebox), and the reaction was carried out overnight at 90 °C under stirring. Then, the modified plate was taken out from the reaction mixture and rinsed with copious amounts of DMF, THF and toluene. After drying under the stream of argon, the sample was ready for the deposition of the next layer. The procedure was repeated alternatingly (donor and acceptor) until the desired number of D-A pairs was deposited. 

### 3.8. Stille Coupling

The reaction mixtures for donor/acceptor deposition steps were prepared in pressure tubes heated under the argon flow. After cooling down to RT, the proper donor or acceptor compound was added: (3,3′-dimethoxy-[2,2′-bithiophene]-5,5′-diyl)bis(trimethylstannane) (**2**, donor) (66 mg, 0.12 mmol) or 4,7-dibromo-5,6-dinitrobenzo[c][1,2,5]thiadiazole (**6**, acceptor) (46 mg, 0.12 mmol), respectively. Next, Pd_2_(dba)_3_ (2.20 mg, 0.0024 mmol) and P(*o*-tol)_3_ (2.92 mg, 0.0096 mmol) were added to the tube and all solids were dissolved in anh. toluene (12 mL). The substrate coated with the initiator monolayer was immersed in the prepared solution (in a glovebox) and the reaction was carried out for 2 h at RT in the case of the donor and overnight at 110 °C for the acceptor reaction under stirring. Then, the modified plate was taken out from the reaction mixture and rinsed with copious amounts of toluene and DCM. After drying under the stream of argon, the sample was ready for deposition of the next layer. The procedure was repeated alternatingly (donor and acceptor) until the desired number of D-A pairs was deposited. 

### 3.9. Click Reaction

The reaction mixtures for donor/acceptor deposition steps were prepared in pressure tubes heated under the argon flow. After cooling down to RT, the proper donor or acceptor compound was added: 3,3′-dimethoxy-5,5′-diethynyl-2,2′-bithiophene (**5**, donor) (33 mg, 0.12 mmol) or 4,7-diazide-5,6-dinitrobenzo[c][1,2,5]thiadiazole (**7**, acceptor) (37 mg, 0.12 mmol), respectively. Next, CuI (2.30 mg, 0.012 mmol) was inserted into the tube and all solids were dissolved in anh. DMSO (12 mL). The substrate coated with the initiator monolayer was immersed in the prepared solution (in a glovebox) and the reaction was carried out for 2 h in the case of the donor and overnight for the acceptor deposition, both at 40 °C and under stirring. Then, the modified plate was taken out from the reaction mixture, and rinsed with copious amounts of DMF, THF and toluene. After drying under the stream of argon, the sample was ready for the deposition of the next layer. The procedure was repeated alternatingly (donor and acceptor) until the desired number of D-A pairs was deposited. 

## 4. Conclusions

We presented an efficient synthetic approach that resulted in the formation of surface-grafted donor-acceptor polymer brushes via stepwise alternating attachment of electron-donating and electron-accepting monomers in a precise manner using selected cross-coupling reactions. Based on the results of the DFT calculations, 3,3′-dimethoxy-2,2′-bithiophene and 5,6-dinitrobenzo[c][1,2,5]thiadiazole were selected as cores of the monomers since they were found to lead to a conjugated polymer with a low optical bandgap. Sonogashira, Stille and Click reactions were applied to form densely grafted chains from novel bifunctionalized monomers. The polymers obtained in the Sonogashira coupling process were shown to form the most uniform and longest molecular wires (thickness of the brushes by ellipsometry - up to ca. 25 nm) due to the presence of a stiff acetylene bridge between the donor and acceptor groups in the chains. The presented methodology allowed us to obtain a uniform organic layer whose thickness can be precisely tuned by varying the number of deposition steps for a given grafting density. The critical role of the appropriate grafting density for efficient stepwise growth of the alternating D-A chains was revealed. Possible limitations are likely related to the inactivation of the growing chains by the formation of loop structures between the neighboring chains during the attachment of bifunctionalized monomers if the grafting density is too large.

The reduced bandgap (Eg ≈ 2.34 eV), as well as the semiconductive character of the polymer brushes, make them a promising system for applications in photovoltaics and optoelectronics. The utilization of other coupling reactions was shown to be feasible, but because of the formation of flexible connections between the mers, the growth of the brushes was less efficient than in the Sonogashira coupling, and the obtained bandgaps were found to be higher in spite of more optimistic results of DFT calculations. The possibility of using various bifunctionalized monomers equipped with either acetylene or halogen substituents makes the proposed approach very versatile and paves the way for syntheses of other donor-acceptor polymer brushes, aiming at even lower optical bandgaps. Moreover, such a macromolecular engineering approach enables the fabrication of more complex copolymer systems with precise control of the sequence of two or more types of mers. 

## Figures and Tables

**Table 1:** Values of calculated HOMO and LUMO levels for various donor and acceptor moieties.

Moiety	HOMO (eV)	LUMO (eV)
**M06-2X**		
2,2′-bithiophene	−6.87387	−2.76604
3,3′-dimethoxy-2,2′-bithiophene	**−6.36066**	0.34776
3,3′-dimethylo-2,2′-bithiophene	−7.04285	0.21334
benzo[c][1,2,5]thiadiazole	−7.96260	−1.37037
5,6-dinitrobenzo[c][1,2,5]thiadiazole	−9.41623	**−2.76604**
**MP4**		
2,2′-bithiophene	−7.84341	1.62126
3,3′-dimethoxy-2,2′-bithiophene	**−7.47987**	1.66425
3,3′-dimethylo-2,2′-bithiophene	−8.06954	1.71922
benzo[c][1,2,5]thiadiazole	−9.00779	0.66369
5,6-dinitrobenzo[c][1,2,5]thiadiazole	−10.67530	**−0.76546**

The highlighted values represent monomers with the best correlation between energy levels.

## Data Availability

All data are contained within the publication or available Supplementary Materials.

## Supplementary Materials

The following supporting information can be downloaded at: https://www.mdpi.com/article/10.3390/ijms23116162/s1. References [57,58] are cited in the supplementary materials.
