# Supplementary material for: Precise Stepwise Synthesis of Donor-Acceptor Conjugated Polymer Brushes Grafted from Surfaces
Source: Int J Mol Sci. 2022 May 31;23(11):6162. doi: 10.3390/ijms23116162 (PMC9181774; doi:10.3390/ijms23116162)
Supplement: Supplementary file 1 [file ijms-23-06162-s001.zip › ijms-1751714-supplementary.pdf]

Precise stepwise synthesis of donor-acceptor conjugated polymer brushes grafted from surfaces

Anna Grobelny [1], Artur Grobelny [2] and Szczepan Zapotoczny [1,*]

[1] Faculty of Chemistry, Jagiellonian University, Gronostajowa 2, 30-387 Kraków, Poland; grobelnyania@gmail.com

[2] Selvita Services Sp. Z o.o., Bobrzyńskiego 14, 30-348 Kraków, Poland; artur.grobelny@selvita.com

* Correspondence: zapotocz@chemia.uj.edu.pl; Tel.: +48-12-686 -25-30

SUPPORTING INFORMATION

Table of content

1. Synthesis of monomers 2

3,3'-dimethoxy-2,2'-bithiophene (**1**) 2

(3,3'-dimethoxy-[2,2'-bithiophene]-5,5′-diyl)bis(trimethylstannane) (**2**) 2

5,5′-dibromo-3,3'-dimethoxy-2,2'-bithiophene (**3**) N 2

3,3'-dimethoxy-5,5′-bis(trimethylsilyl)ethynyl-2,2′-bithiophene (**4**) N 2

5,5′-diethynyl-3,3'-dimethoxy-2,2′-bithiophene (**5**) N 2

4,7-dibromo-5,6-dinitrobenzo[c][1,2,5]thiadiazole (**6**) 3

4,7-diazide-5,6-dinitrobenzo[c][1,2,5]thiadiazole (**7**) N 3

2. Estimation of molar extinction coefficient 3

3. Estimation of conductivity 3

4. Supplementary Figures 4

1. Synthesis of monomers

All reactions were conducted under argon atmosphere. Compounds signed as ^{N}, to the best of our knowledge, have not been reported in the literature so far, (based on Sci-Finder® and Reaxys® data base).

*3,3′-dimethoxy-2,2′-bithiophene (**1**)*

3-methoxythiophene (10 g, 87.6 mmol) was dissolved in anhydrous THF (100 ml), cooled to -78°C and 2.5 M *n*-BuLi in hexane (35 ml, 87.6 mmol) was added dropwise. The reaction mixture was stirred for 2 h. Then, Fe(acac)$_3$ (46.4 g, 131.4 mmol) was added portionwise. The solution was stirred overnight at RT and neutralized with small amount of MeOH. The red precipitate was filtered, washed with DCM and filtrate was concentrated *in vacuo*. The obtained residue was purified by column chromatography (silica gel, hexane:ethyl acetate | 1:0 → 96:4), followed by trituration in hexane to afford product **1** as a beige solid (4.3 g, 44%).

^{1}H NMR (400 MHz, CDCl$_3$, δ): 7.11 (d, *J* = 5.5 Hz, 2H), 6.87 (d, *J* = 5.5 Hz, 2H), 3.95 (s, 6H). ^{13}C NMR (100 MHz, CDCl$_3$, δ): 152.9, 122.0, 115.8, 113.8, 59.0.

*(3,3′-dimethoxy-[2,2′-bithiophene]-5,5′-diyl)bis(trimethylstannane) (**2**)*

Compound **2** was synthesized according to the procedure reported in the literature. [1] Briefly, the solution of 3,3'-dimethoxy-2,2'-bithiophene 1 (0.40 g, 1.8 mmol) in anh. THF (8 ml) was cooled to -78°C and 2.5 M *n*-BuLi in hexane (2.8 ml, 7.1 mmol) was added dropwise. After 15 min of stirring, the reaction mixture was heated up to RT and cooled down to -78°C again. Then, the solution of Me$_3$SnCl (1.4 g, 7.1 mmol) in anh. THF (7 ml) was added dropwise. The reaction was left to proceed overnight, diluted with water and extracted with Et$_2$O (1×50 ml). Obtained organic layer was washed with water (3 × 50 ml), dried over anh. Na$_2$SO$_4$ and concentrated *in vacuo.* The crude material was next recrystallized from MeCN to get the final compound **2** as white crystals (0.71 g, 73%).

^{1}H NMR (400 MHz, CDCl$_3$, δ): 6.91 (s, 2H), 3.97 (s, 6H), 0.37 (s, 18H). ^{13}C NMR (100 MHz, CDCl$_3$, δ): 154.7, 134.2, 123.4, 119.8, 59.2, -8.1.

HRMS (APCI$^+$): [M+H]$^+$ calculated for $C_{16}H_{27}O_2S_2Sn_2^+$: 554.9491; found: 554.9466. HRMS (APCI$^-$): no ionization.

*5,5′-dibromo-3,3′-dimethoxy-2,2′-bithiophene (**3**)* ^{N}

3,3'-dimethoxy-2,2'-bithiophene **1** (0.50 g, 2.2 mmol) was dissolved in anh. THF (10 ml) and NBS (0.79 g, 4.4 mmol) was added portionwise without access to the light. After 10 min of stirring at RT the reaction mixture was neutralized with sat. Na$_2$S$_2$O$_3$ (10 ml) and diluted with water. The solution was extracted with DCM (3×25 ml). All organic layers were combined, dried over anh. Na$_2$SO$_4$ and concentrated *in vacuo.* The crude material was purified *via* column chromatrography (Al$_2$O$_3$, hexane:ethyl acetate | 1:0 → 1:1) to obtain a target compound **3** as a white solid (0.77 g, *quant.*). ^{1}H NMR (300 MHz, CDCl$_3$, δ): 6.84 (s, 2H), 3.90 (s, 6H). ^{13}C NMR (75 MHz, CDCl$_3$, δ): 151.4, 118.9, 115.1, 110.3, 59.3.

*3,3′-dimethoxy-5,5′-bis(trimethylsilyl)ethynyl-2,2′-bithiophene (**4**)* ^{N}

5,5′-dibromo-3,3'-dimethoxy-2,2'-bithiophene **3** (1.6 g, 4.2 mmol) was dissolved in mixture of anh. THF (40 ml) and *i*-Pr$_2$NH (20ml) in a pressure tube and next trimethylsilylacetylene (4.1 g, 5.9 ml, 41.7 mmol), Pd(PPh$_3$)$_4$ (0.24 g, 0.2 mmol) and CuI (40.0 mg, 0.2 mmol) were sequentially added. The reaction mixture was heated up to 60 °C and stirred overnight. Next, the solution was quenched with water and extracted with CHCl$_3$ (3×100 ml). The combined organic layers were dried over anh. Na$_2$SO$_4$ and concentrated *in vacuo.* The residue was purified by column chromatography (silica gel, hexane:ethyl acetate | 1:0 → 98:2) to afford desired molecule **4** as brownish solid (1.6 g, 94%).

^{1}H NMR (300 MHz, CDCl$_3$, δ): 6.97 (s, 2H), 3.92 (s, 6H),0.25 (s, 18H). ^{13}C NMR (75 MHz, CDCl$_3$, δ): 152.3, 120.2, 119.1, 115.9, 100.3, 98.5, 59.0, 0.0.

*5,5′-diethynyl-3,3′-dimethoxy-2,2′-bithiophene (**5**)* ^{N}

K$_2$CO$_3$ (0.23 g, 1.7 mmol) was added portionwise to the solution of 3,3'-dimethoxy-5,5′-bis(trimethylsilyl)ethynyl-2,2′-bithiophene **4** (0.70 g, 1.7 mmol) in MeOH (35 ml). The reaction mixture was stirred at RT for 4 h. The solution was diluted with water and extracted with DCM (3×20 ml). All organic layers were combined, dried over anh. Na$_2$SO$_4$ and concentrated *in vacuo.* The crude material was purified by trituration in hexane to obtain final monomer **5** as a brownish solid (0.54 g, 85%).

^{1}H NMR (400 MHz, CDCl$_3$, δ): 7.02 (s, 2H), 3.94 (s, 6H), 3.42 (s, 2H). ^{13}C NMR (75 MHz, CDCl$_3$, δ): 152.3, 120.7, 118.1, 116.0, 82.5, 77.8, 59.1.

HRMS (APCI$^+$): [M+H]$^+$ calculated for $C_{14}H_{11}O_2S_2^+$: 275.0195; found: 275.0196. HRMS (APCI$^-$): [M-H]$^-$ calculated for $C_{14}H_9O_2S_2^-$: 273.0049; found: 273.0017.

*4,7-dibromo-5,6-dinitrobenzo[c][1,2,5]thiadiazole (**6**)*

Molecule **6** was synthesized according to the procedure reported in the literature. [2] Briefly, 4,7-dibromo-benzo[c][1,2,5]thiadiazole (3.0 g, 10.2 mmol) was added portionwise to fuming CF_3SO_3H (45 ml) in 0°C, followed by dropping of fuming HNO_3 (45 ml). The reaction mixture was stirred overnight at RT, poured into ice water and left for crystallization for 30 min. The obtained crude product was filtered, washed several times with water and purified *via* flesh column chromatography (silica gel, hexane:DCM | 1:1) to afford product **6** as a yellow solid (3.5 g, 89%).
^{13}C NMR (75 MHz, DMSO-d_6, δ): 151.5, 143.6, 111.3.
HRMS (APCI$^+$): no ionization. HRMS (APCI$^-$): $[M]^-$ calculated for $C_6Br_2N_4O_4S^-$: 381.8012; found: 381.8010.

*4,7-diazide-5,6-dinitrobenzo[c][1,2,5]thiadiazole (**7**)* [N]

4,7-dibromo-5,6-dinitrobenzo[c][1,2,5]thiadiazole **6** (0.20 g, 0.5 mmol) was dissolved in DMF (2 ml) and the mixture was cooled down to 0°C. Then the solution of NaN_3 (74.0 mg, 1.1 mmol) in water (0.7 ml) was added dropwise. The reaction was left to proceed for 1 h at RT and it was poured into ice water. The obtained product was washed with water several times and dried *in vacuo* to get target molecule **7** as a yellow solid (0.12 g, 72%). Due to instability of the compound, it was freshly prepared before each process of polymer brushes synthesis.
^{13}C NMR (75 MHz, DMSO-d_6, δ): 148.7, 131.7, 124.0.
HRMS (ESI$^+$): no ionization.HRMS (ESI$^-$): no ionization.

2. Estimation of molar extinction coefficient

The value of molar extinction coefficient (α) was estimated from recorded UV-Vis spectra according to the commonly known formula:

$$\alpha = 2.303\frac{A}{l}$$

where:
A – absorbance
l – optical path length, determined as brushes thickness given in cm

3. Estimation of conductivity

The value of the conductivity (κ) was estimated according to the formula:

$$\kappa = \frac{L}{R \cdot S} = \frac{I}{U} \cdot \frac{L}{2\pi rh}$$

where:
L = 13.9 nm – thickness of polymer brushes
R – electrical resistance of polymer brushes
S – contact area between the tip and polymer brushes
$\frac{I}{U} = 200\ \frac{pA}{V}$ – the slope of I/V curves collected in the range from -3V to -4V
r = 133 nm – tip radius
h = 4 nm - tip indentation into polymer material

4. Supplementary Figures

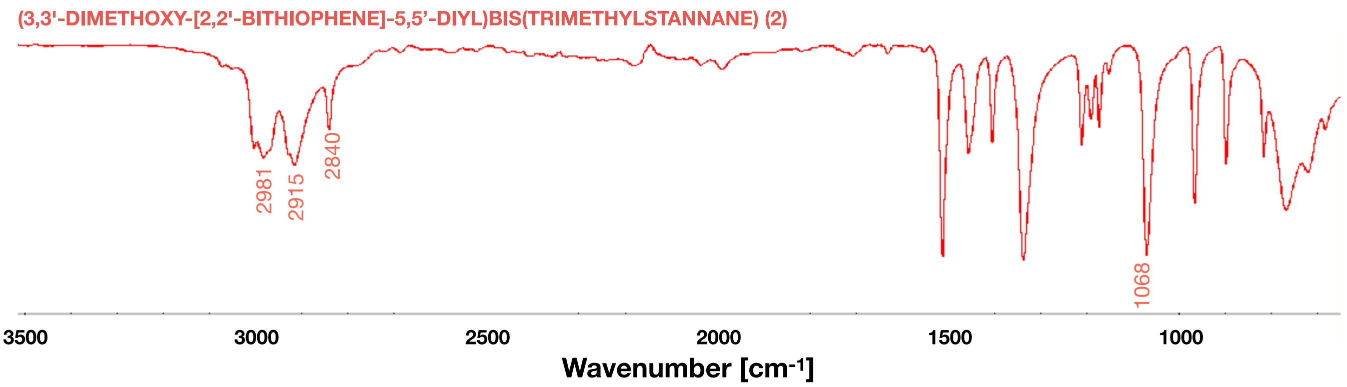

Figure S1. FTIR spectrum of (3,3'-dimethoxy-[2,2'-bithiophene]-5,5′-diyl)bis (trimethylstannane) (**2**):
1068 cm^{-1} (C-O stretching vibrations in methoxy group), 2840, 2915 and 2981 cm^{-1} (C-H stretching vibrations in $Sn(CH_3)_3$ group).

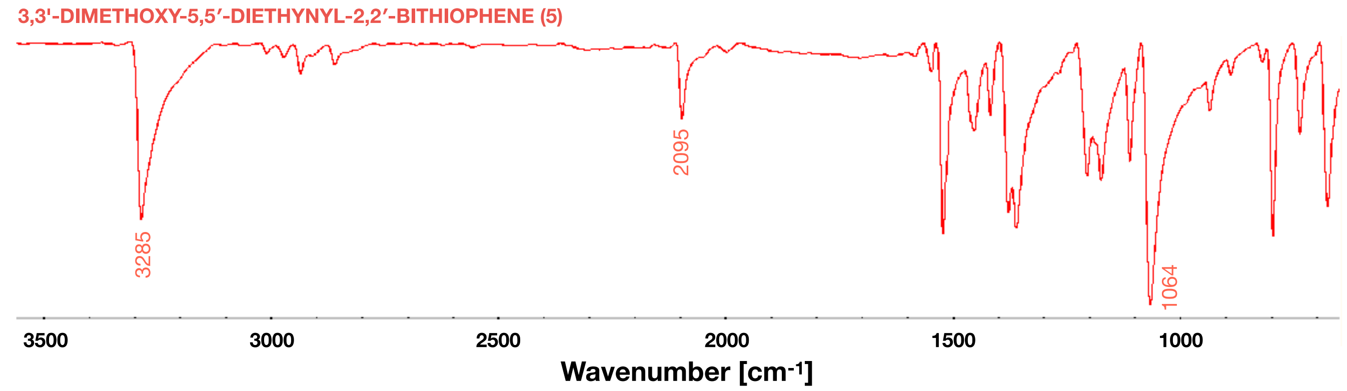

Figure S2. FTIR spectrum of 3,3'-dimethoxy-5,5′-diethynyl-2,2′-bithiophene (**5**):
1064 cm^{-1} (C-O stretching vibrations in methoxy group), 2095 cm^{-1} (C≡C stretching vibration), 3285 cm^{-1} (C-H stretching vibrations in acetylene group).

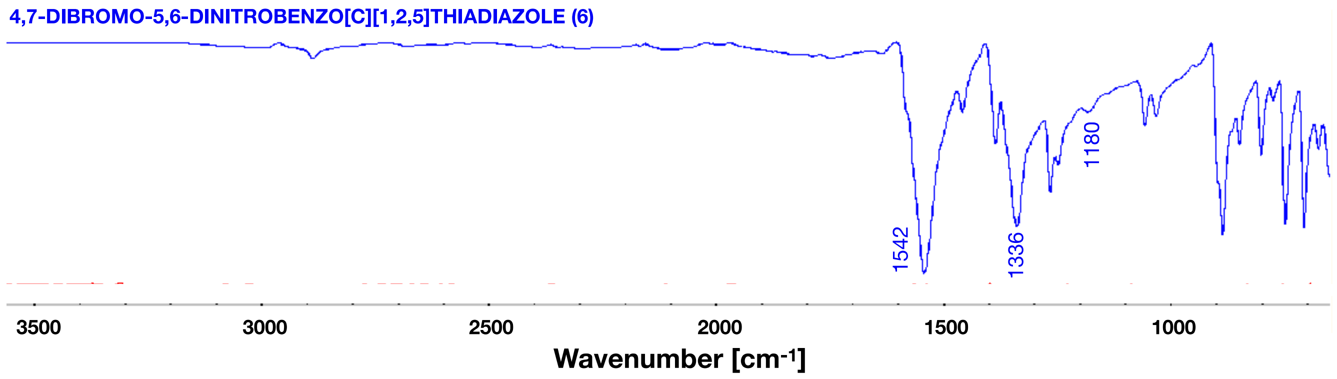

Figure S3. FTIR spectrum of 4,7-dibromo-5,6-dinitrobenzo[c][1,2,5]thiadiazole (**6**):
1180 cm^{-1} (C_{Ar}-Br stretching vibrations), 1336 and 1542 cm^{-1} (N=O stretching vibrations in $-NO_2$ group).

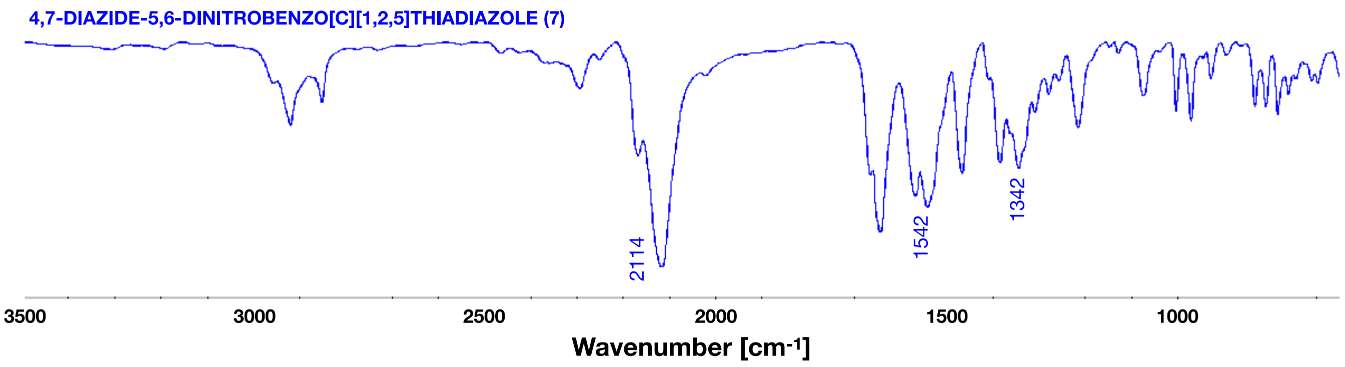

Figure S4. FTIR spectrum of 4,7-diazide-5,6-dinitrobenzo[c][1,2,5]thiadiazole (**7**): 1342 and 1542 cm^{-1} (N=O stretching vibrations in -NO_2 group), 2114 cm^{-1} (N=N=N stretching vibrations in azide group).

(A)

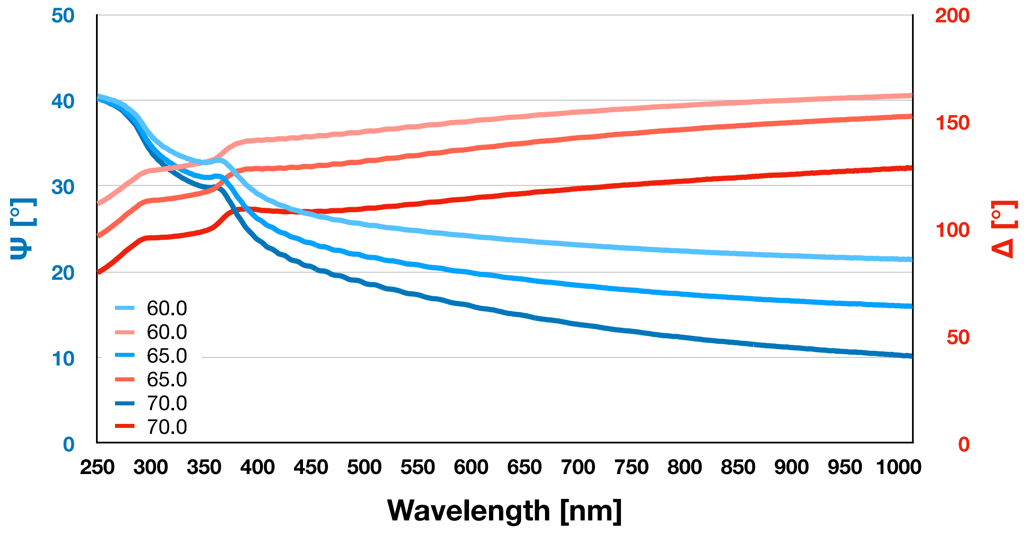

(B)

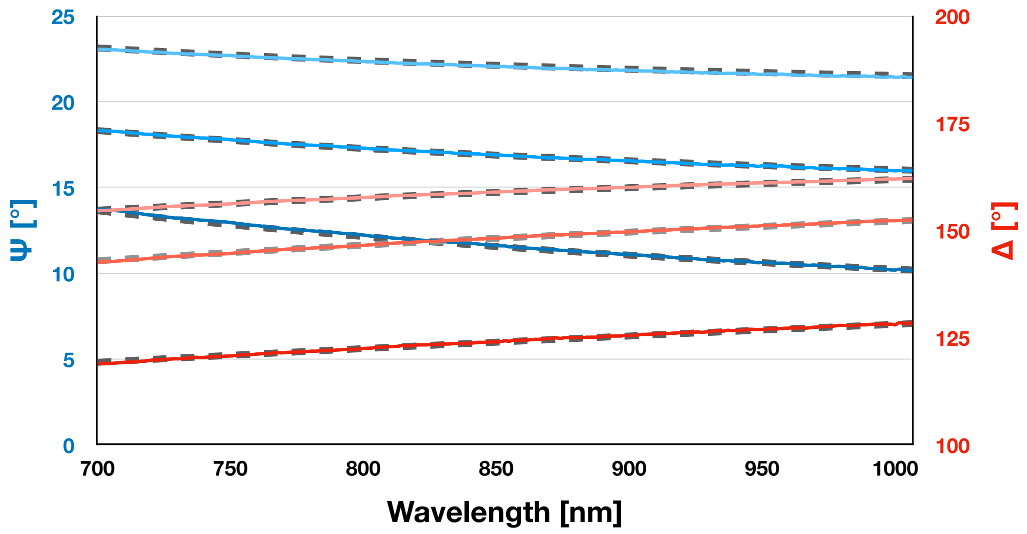

Figure S5. Exemplary spectroscopic ellipsometry data obtained for the brushes prepared *via* Sonogashira coupling on silica substrate (**a**) (at angels 60°, 65° and 70°), together with their fits to the Cauchy model (**b**).

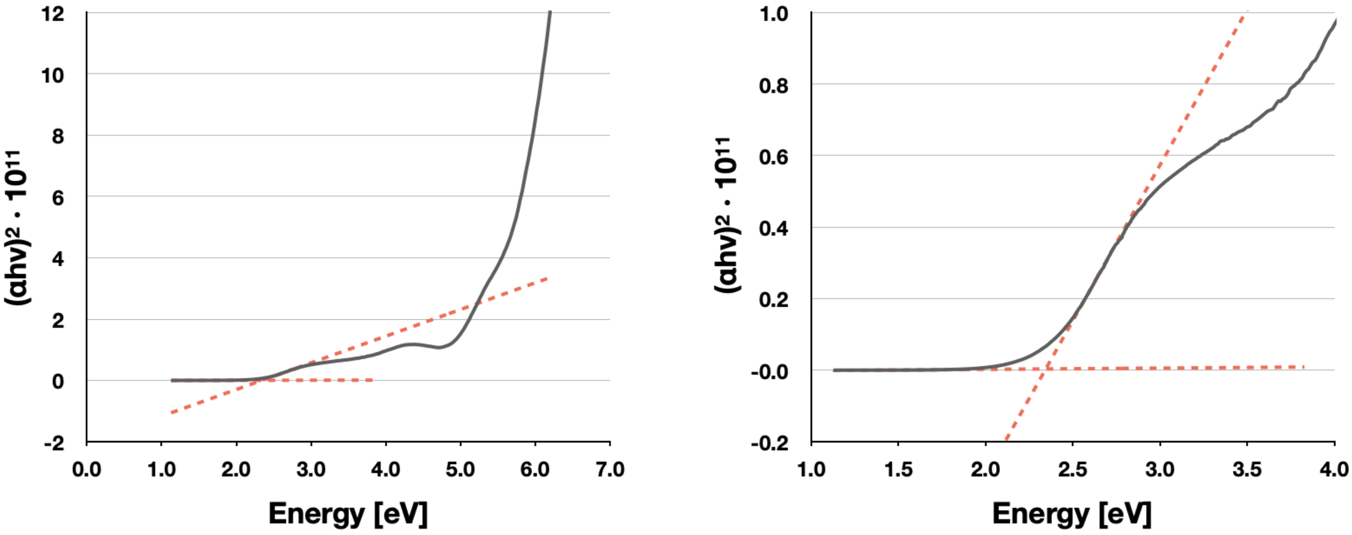

Figure S6. Direct bandgap determination of polymer brushes obtained *via* Sonogashira coupling on quartz substrate using UV-Vis spectra applying the Tauc plot method.

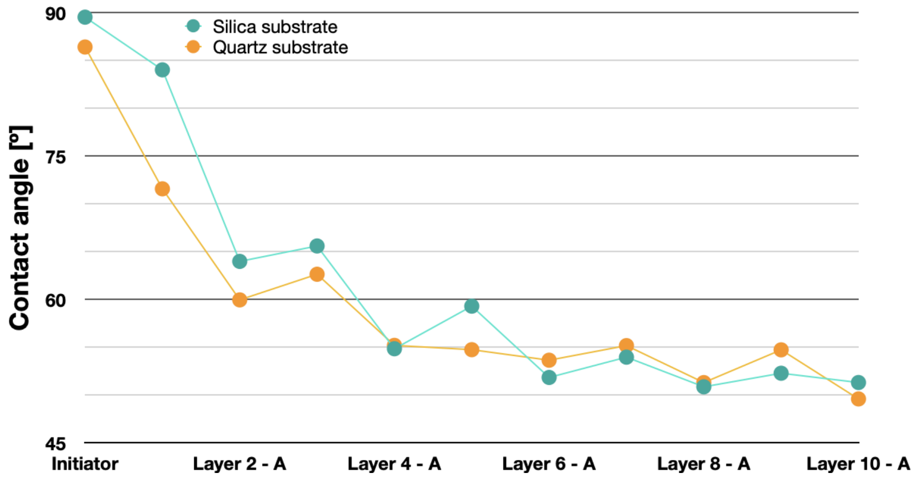

Figure S7. Contact angle measurements of the layers obtained *via* Sonogashira coupling reaction.

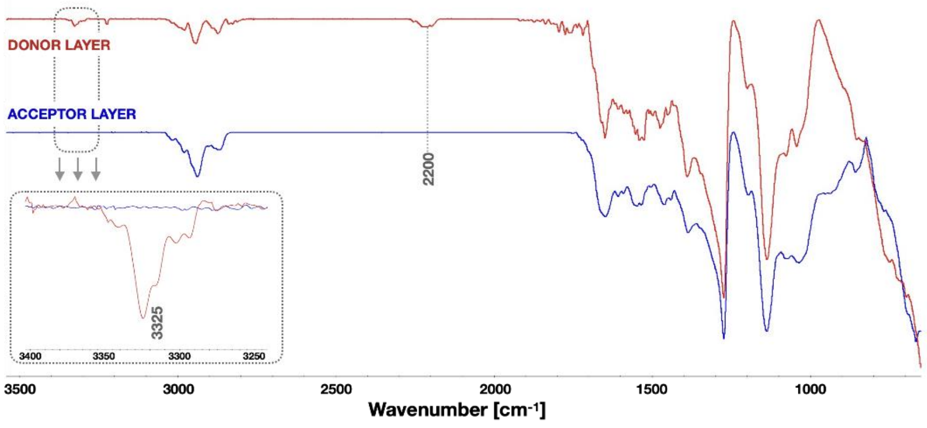

Figure S8. The transmittance IR spectra registered after a single donor and subsequent acceptor coupling on ITO surface *via* Sonogashira coupling reaction.

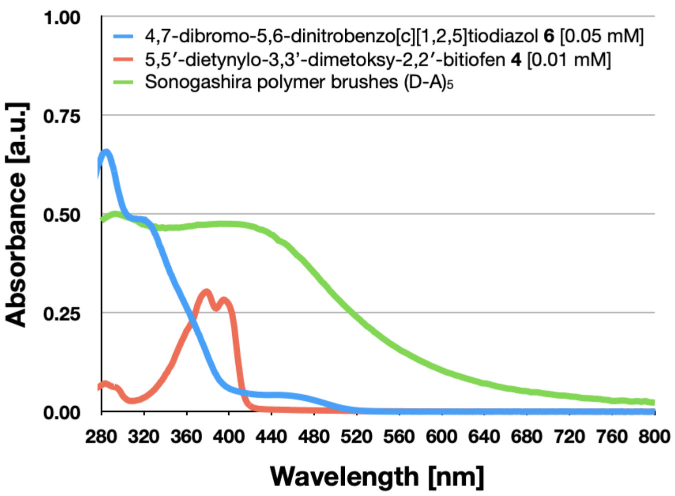

Figure S9. UV-Vis spectra of the monomers used to synthesis of donor-acceptor polymer brushes *via* Sonogashria coupling. Solutions of both compounds are prepared in toluene. The spectra of polymer brushes were normalized to absorbance of 0.5.

Table S1. Ratios of atomic concentrations (N:Cl) in the mixed APTES/ClPTES monolayers on silica substrate based on XPS measurements and the calculated surface grafting density of the active species.

APTES/ClPTES feed ratio	N:Cl in the monolayer	Surfaces concentration of APTES (grafting density)
25%	0.6	40%
50%	2.2	70%
75%	4.5	80%

[1] full APTES monolayer

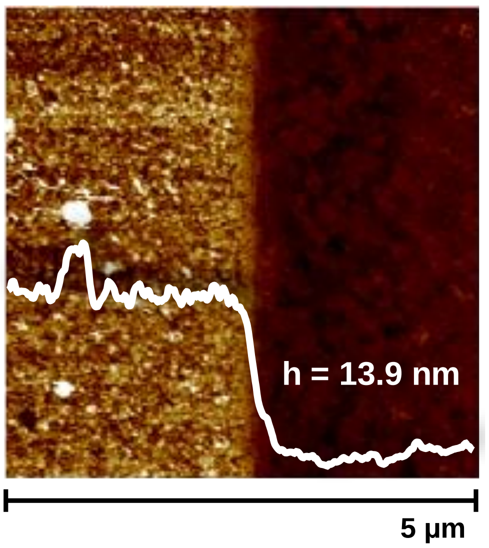

Figure S10. AFM image visualizing thickness of polymer brushes obtained *via* Sonogashira coupling on ITO substrate and used for the conductivity measurements.

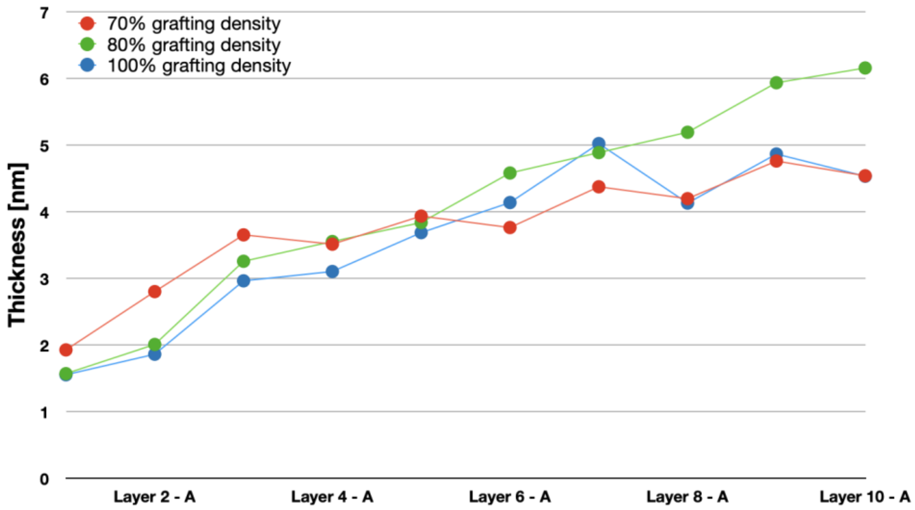

Figure S11. Thickness of D-A polymer brushes with different grafting density obtained *via* Stille coupling reaction on silica substarte, as determined by spectroscopic ellipsometry.

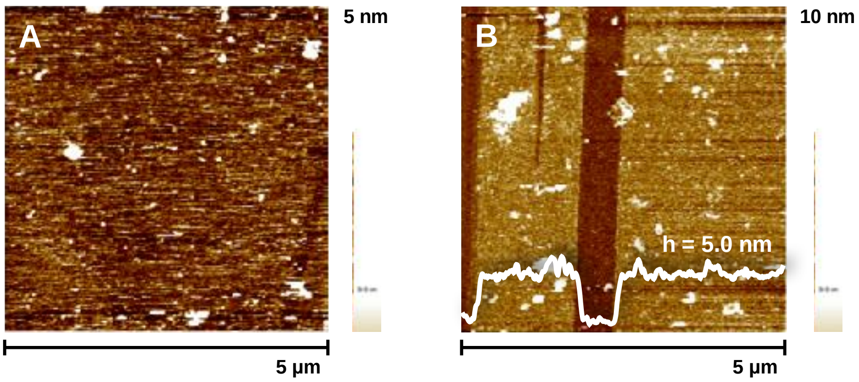

Figure S12. AFM images of polymer brushes obtained *via* Stille coupling on silica substrate: (**a**) – topography, (**b**) – after deposition of 10 layers.

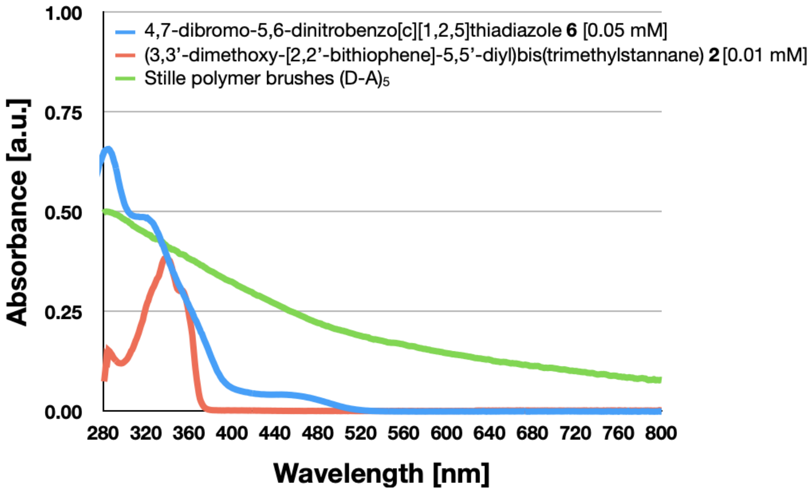

Figure S13. UV-Vis spectra of the monomers used to synthesis of donor-acceptor polymer brushes *via* Stille reaction.
Solutions of both compounds are prepared in toluene. The spectra of polymer brushes were normalized to absorbance of 0.5.

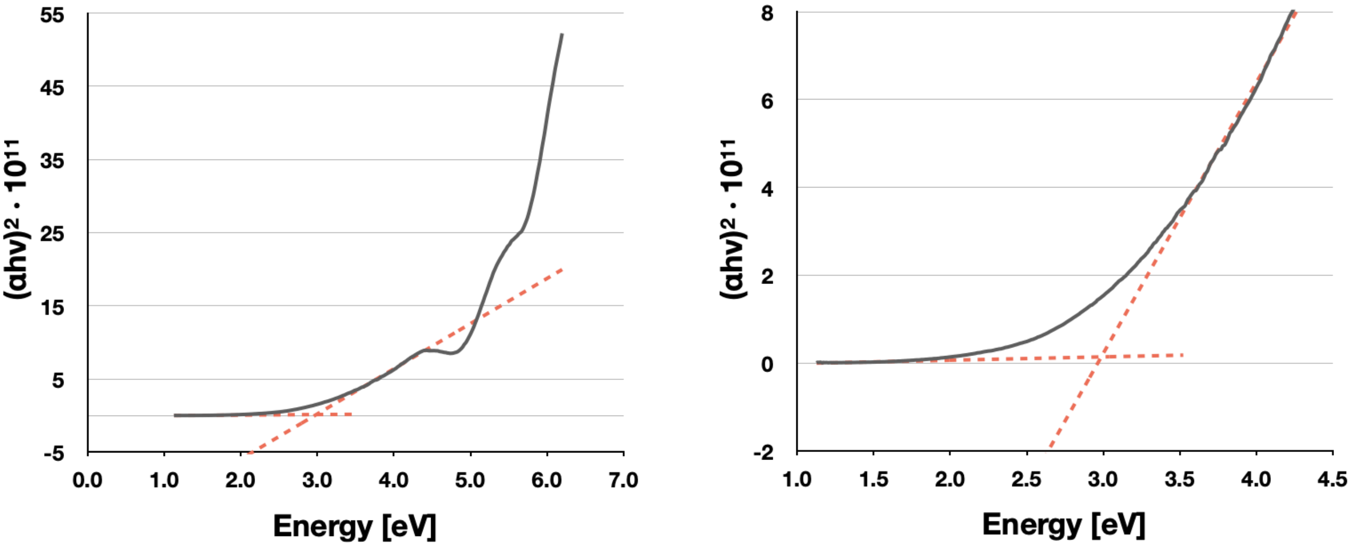

Figure S14. Direct bandgap determination of polymer brushes obtained *via* Stille coupling on quartz substrate using UV-Vis spectra applying the Tauc plot method.

Table S2. Atomic concentrations of Cl (based on XPS measurements) in the mixed monolayers on silica substrate containing active (*p*-chloromethyl)phenyltrimethoxysilane and inactive trimethoxy(*p*-tolyl) silane and the calculated surface grafting density of the active species.

(p-chloromethyl)phenyltrimethoxysilane content in the feed mixture	% Cl	Surface grafting density
50%	1.54	70%
75%	1.69	80%
100%	2.14	100%[1]

[1] full (*p*-chloromethyl)phenyltrimethoxysilane monolayer

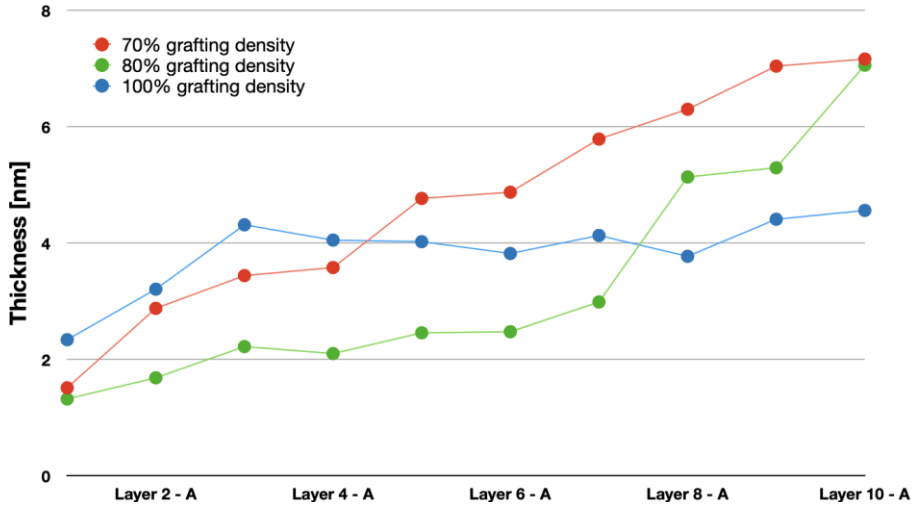

Figure S15. Thickness of D-A polymer brushes with different grafting density obtained *via* Click reaction on silica substrate, as determined using spectroscopic ellipsometry.

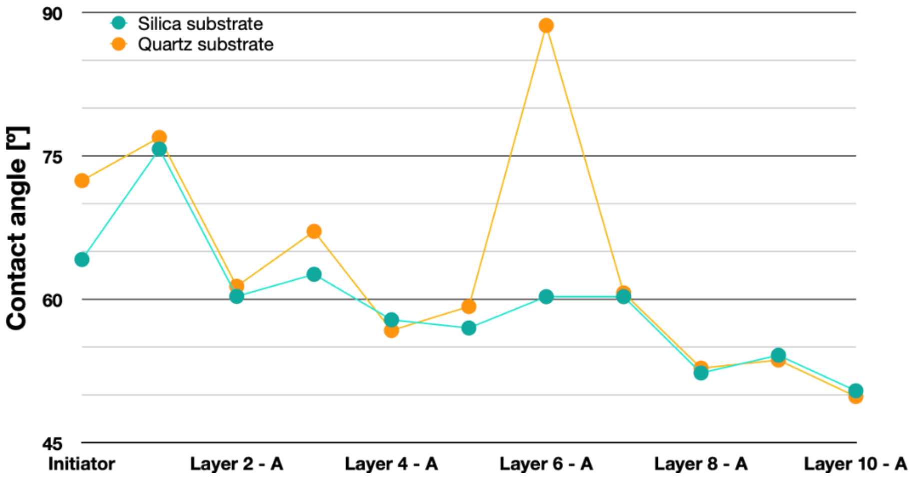

Figure S16. Contact angle measurements of the layers obtained *via* Click reaction.

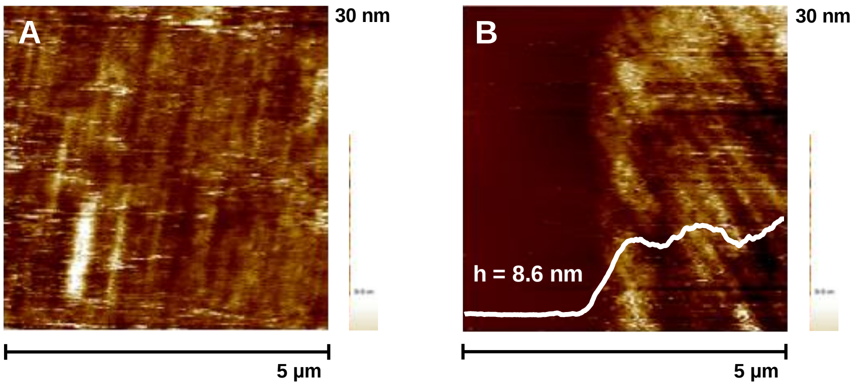

Figure S17. AFM images of polymer brushes obtained *via* Click reaction on silica substrate: (**a**) – topography, (**b**) – thickness after deposition of 10 layers.

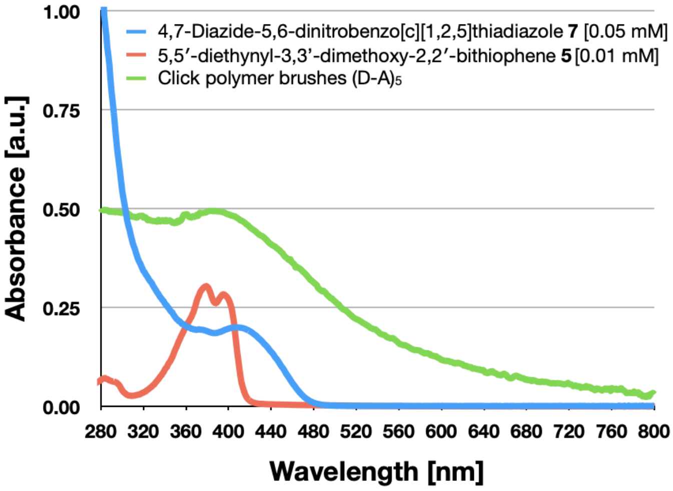

Figure S18. UV-Vis spectra of the monomers used to synthesis of donor-acceptor polymer brushes *via* Click reaction. Solutions of both compounds are prepared in toluene. The spectra of polymer brushes were normalized to absorbance of 0.5.

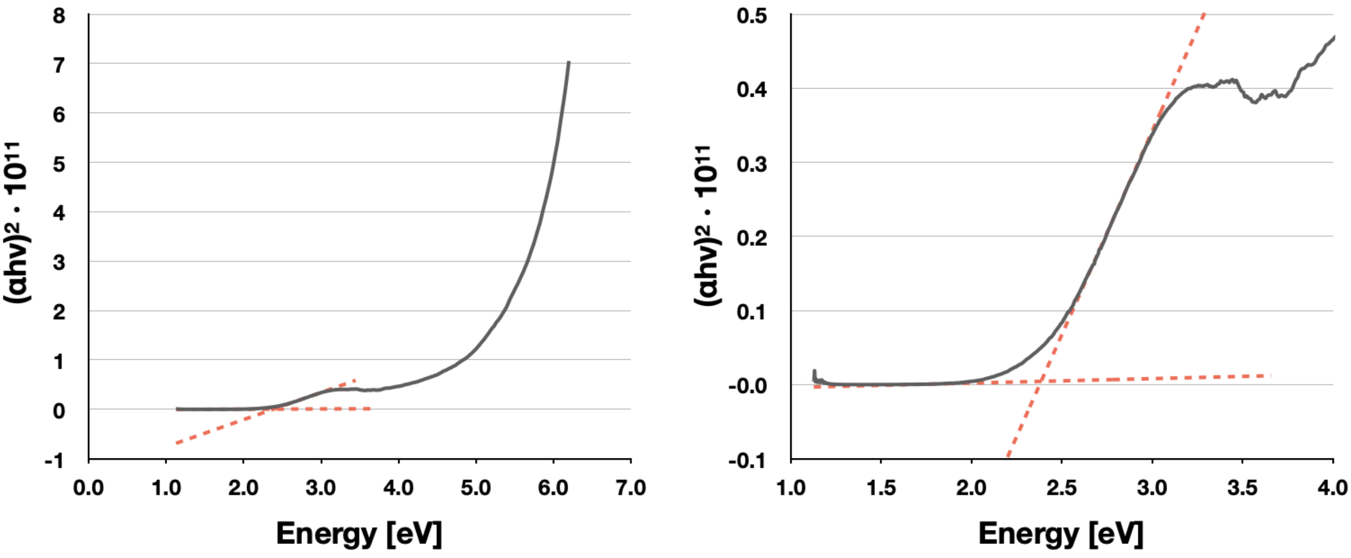

Figure S19. Direct bandgap determination of polymer brushes obtained *via* Click reaction on quartz substrate using UV-Vis spectra applying the Tauc plot method.

1. Giovannitti, A.; Thorley, K. J.; Nielsen, C. B.; Li, J.; Donahue, M. J.; Malliaras, G. G.; Rivnay, J.; McCulloch, I. Redox-Stability of Alkoxy-BDT Copolymers and their Use for Organic Bioelectronic Devices. *Advanced Functional Materials*, **2018**, *28*, 1706325, doi:10.1002/adfm.201706325.
2. Hassan Omar, O.; la Gatta, S.; Tangorra, R. R.; Milano, F.; Ragni, R.; Operamolla, A.; Argazzi, R.; Chiorboli, C.; Agostiano, A.; Trotta M.; Farinola, G. M. Synthetic Antenna Functioning As Light Harvester in the Whole Visible Region for Enhanced Hybrid Photosynthetic Reaction Centers. *Bioconjugate Chemistry*, **2016**, *27*, 1614–1623, doi:10.1021/acs.bioconjchem.6b00175.
